# Neural and Hormonal Basis of Opposite-Sex Preference by Chemosensory Signals

**DOI:** 10.3390/ijms22158311

**Published:** 2021-08-02

**Authors:** Yasuhiko Kondo, Himeka Hayashi

**Affiliations:** Department of Animal Sciences, Faculty of Life and Environmental Science, Teikyo University of Science, Uenohara 409-0193, Yamanashi, Japan; g20md004@st.ntu.ac.jp

**Keywords:** sexual preference, pheromones, olfactory epithelium, vomeronasal organ, sex steroids, olfactory nervous system, sexual experience, neuropeptide

## Abstract

In mammalian reproduction, sexually active males seek female conspecifics, while estrous females try to approach males. This sex-specific response tendency is called sexual preference. In small rodents, sexual preference cues are mainly chemosensory signals, including pheromones. In this article, we review the physiological mechanisms involved in sexual preference for opposite-sex chemosensory signals in well-studied laboratory rodents, mice, rats, and hamsters of both sexes, especially an overview of peripheral sensory receptors, and hormonal and central regulation. In the hormonal regulation section, we discuss potential rodent brain bisexuality, as it includes neural substrates controlling both masculine and feminine sexual preferences, i.e., masculine preference for female odors and the opposite. In the central regulation section, we show the substantial circuit regulating sexual preference and also the influence of sexual experience that innate attractants activate in the brain reward system to establish the learned attractant. Finally, we review the regulation of sexual preference by neuropeptides, oxytocin, vasopressin, and kisspeptin. Through this review, we clarified the contradictions and deficiencies in our current knowledge on the neuroendocrine regulation of sexual preference and sought to present problems requiring further study.

## 1. Introduction

While short-lived microbes have high mutation rates to obtain evolutional benefits against environmental pressure, long-life organisms, such as mammals, use diversity. This intraspecific diversity is achieved by sexual reproduction to disperse genes within conspecifics. To enable sexual reproduction, organisms have sexually differentiated their reproductive roles, resulting in the requirement of interaction with the opposite sex. To successfully breed offspring, animals, particularly small mammals, have evolved systems to find, attract, and approach the opposite sex, by chemosensory signals called pheromones.

The first attempts of systematic behavioral testing for opposite-sex selection or preference in laboratory rodents were reported by Slob et al. [1,2,3,4,5]. To determine the conspecifics an individual is attracted to, while simultaneously presenting various types of stimulus animals, it is possible to measure which animals the test subjects try to approach, investigate, and make contact with. The paired stimuli for simultaneous presentation would be opposing, such as estrous females vs. sexually active males, estrous females vs. anestrous females, and sexually active males vs. castrated males. In early studies, free moving rats were tested in a three-chamber apparatus with side chambers containing male and female stimulus rats unable to move beyond their chamber. In this context, male rats showed an opposite-sex preference by spending longer time in the chamber with estrous females than in that of males (same-sex) [4]. For female rats, however, it was more complicated. Insofar as males can copulate, receptive females prefer sexually inactive males rather than active ones [2], but when direct interactions were blocked by a wire-mesh barrier between them [1] or intromissions were prevented by vaginal occlusion [2], the receptive females showed a clear preference to spend longer time in the vicinity of sexually active male rats.

**Figure 1:**
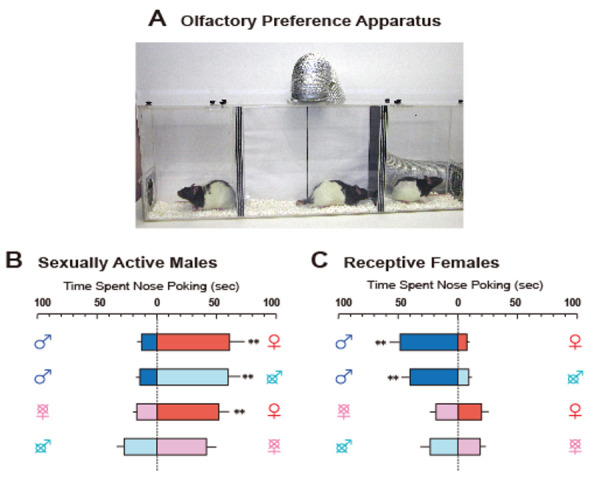
(**A**). The apparatus for olfactory preference test in rats, consisting of three compartments. Experimental rats were placed in the middle, and stimulus rats were placed in two neighboring side compartments. A fan connecting to the airduct at the ceiling of the middle compartment dispersed airflow from the two side compartments to convey the odors of stimulus rats. A transparent tube was placed on each air inlet in the middle compartment to facilitate observation of nose-poking. (**B**) and (**C**). Typical olfactory preference shown by sexually active male rats (**B**) and receptive female rats (**C**). Sex symbol characters on both sides of each column indicate the type of stimulus rat. Crosses over the sex symbol characters indicate gonadectomy of stimulus rat [6]. Reprinted with permission from Ref. [6]. Copyright 2004 Elsevier.

Therefore, sexual partner preferences, which appear to be simple phenomena, have been found to be influenced by many factors, and do not respond to a simple model of sexually dimorphic brain function. Therefore, we sought to develop a preference test for opposite-sex odors limited to airborne chemicals, components of sexual stimuli [6]. Figure 1A depicts our preference test apparatus in rats. The apparatus was divided into three chambers by spaced triplicate boards with a hole at different levels that enabled airflow. A fan connected to the ceiling of the center chamber helped move the airflow carrying the odors from the stimulus animals in the side chambers to subject rats in the center one. Odors led to subjects poking their noses into the air inlet to investigate them (for easier observation of nose-poking behavior, a transparent tube was attached to the inlets). This apparatus allowed us to obtain clear data on sex-specific odor preference patterns (Figure 1B,C). In this paper, we review recent findings on neural and hormonal preference regulation for conspecific odors in laboratory rodents, mice, rats, and hamsters.

## 2. Detection of Sexual Chemicals

The rodent nasal cavity has mainly two chemosensory sensory organs, the vomeronasal organ (VNO) and the olfactory epithelium (OE). However, recently, another chemosensory receptor in the end of the mouse nasal cavity, the Grueneberg ganglion, has been reported. It detects alarm pheromones and predator odors [7,8,9]. The VNO hitherto had been thought to be a chemical detector specialized for pheromones, and the OE a detector of airborne chemicals, handling the role of general olfaction. Furthermore, these receptors’ ligands have almost no overlap; olfactory neurons in the OE express a single type of G-protein-coupled receptor (GPCR), that is, detecting a single ligand, whereas some vomeronasal neurons in the VNO express multiple GPCR types, detecting multiple ligands [10].

Vomeronasal neurons express two classes of receptors: the V1R coupled with Gi2α and the V2R coupled with Goα proteins. In the mouse, vomeronasal sensory epithelium, neurons with V1R in the apical layer project their axons to the rostral part of the accessory olfactory bulb (AOB) and detect small, volatile molecules, while V2R, in the basal layer, project to the caudal AOB region and detect larger, nonvolatile molecules such as peptides and proteins. It has been reported that the use of these vomeronasal receptors differs between males and females [11]. Exposure to soiled bedding collected from sexually active males activates neurons with V2R in the basal layer in estrogen-primed female mice, whereas exposure to estrous female soiled bedding activates neurons with both V1R and V2R in both apical and basal layers in males, but not in estrogen-primed females. Thus, sex differences in V1R and V2R reactivity to the same social chemicals in the peripheral VNO may exist.

Sulfated estrogens are detected as pheromones by V1rj, a V1R subclass, and other unknown molecules in female mouse urine are detected by the V1re and V1rj subclasses, although each one alone has no effect on male mouse behavior. When combined, however, they strongly induce male courtship behavior in mice [12]. On the other hand, darcin (also known as a major urine protein, MUP20), a V2R ligand contained in male mouse urine that provokes aggressive behavior in male mice [13,14], can strongly attract receptive females to the intact male urine [15,16]. In addition, darcin also acts as a reinforcer; that is, when associated with airborne neutral odors in the urine, it produces the learned attraction to male odors in receptive females [15,16]. Exocrine gland-secreting peptide 1 (ESP1) has also been found in the male lacrimal gland as a V2R ligand [17]. Although it has been reported that contact with male mouse pheromone ESP1 enhanced lordosis behavior in receptive females [17], it is still uncertain whether this nonvolatile chemical contributes to the male sexual attraction of receptive female mice. Interestingly, proteome analysis in wild mice (*Mus musculus*) showed darcin and ESP1 also in saliva [18].

Though olfactory neurons express >900 canonical olfactory receptors in the mouse OE [19], trace amino-associated receptors (TAARs) have received attention as noncanonical olfactory receptors for pheromonal signals [20,21]. All TAARs found in 15 mouse genes and 17 genes in the rat function as olfactory receptors, except for TAAR1 [22]. Deficiency of TAARs 2–9 in male mice has been reported to abolish approach behavior toward receptive females [23], while exposure to isobutylamine, a volatile urinary amine considered to be a TAAR3 ligand, increased the activity in the brain regions related to male sexual behavior in wild type (WT) but not in TAARs 2–9 knock-out (KO) mice [23]. Trimethylamine, a TAAR5 ligand, abundantly contained in male mouse urine (>1000 times that of rat urine), works as an incentive for receptive female mice (but as a repellent for rats). TAAR5-KO females do not approach male mouse urine or trimethylamine [24]. All olfactory neurons expressing TAARs in the OE project their axons to specific (necklace) glomeruli surrounding the main olfactory bulb (MOB) [25,26] specialized in processing biologically significant signals, such as social cues, edible and rotten foods, and predator odors [27].

In addition, some epithelial neurons with canonical olfactory receptors may detect pheromones or other social signals. In fact, epithelial neurons express canonical olfactory receptors and transient receptor potential C2 (TRPc2), similar to VNO sensory neurons with V1R or V2R [28]. At present, the function of these neurons is unknown. What types of stimuli these neurons are sensing, and how they are involved in the perception of opposite-sex odors, are questions that warrant further research.

## 3. Sexual Stimuli Attributes

The studies on sensory receptors and their ligands, such as mentioned above, remind us that, while studying sexual preference behavior, we should always be aware of the attributes of the stimuli presented to experimental animals. This is because many studies on sexual preference often use a complex of natural stimuli rather than a single chemical as experimental stimulus. Early studies reported that the odor of the homogenized preputial gland attracts the opposite sex in rats [29] and mice [30]. In a later study in rats, a mixture of squalene extracted from the preputial gland and combined with 2-heptanone and 4-ethyl phenol extracted from male urine was attractive for female rats [31].

Rodent urine contains MUPs. While most mammals studied, such as pigs, cattle, cats, and dogs, have only a single MUP (humans have none), rodents have >20 genes encoding MUPs [32]. Some are highly diverse and used for social recognition as individual volatile odor signatures [33]. On the other hand, some MUPs directly act on vomeronasal receptors as pheromones, promoting puberty onset (Vandenbergh effect, [34]), inducing estrus in cyclic females (Whitten effect, [35]), etc. These pheromones have relatively high molecular weights and are nonvolatile, so they require direct nose contact to transmit the signals [36,37]. Furthermore, MUPs not only function as pheromones, but some act as pheromone transporter molecules [38,39,40]. For example, 2-sec-butyl-4,5-dihydrothiazole and 3,4-deydro-exo-brevicomin are transported by binding a MUP pocket, and cooperatively attract receptive female mice, although none is effective on its own [41,42].

In many studies examining sexual preference, soiled beddings collected from home cages of estrous females and sexually active males have been used as paired stimuli [43,44,45,46,47]. In such tests, however, it is not possible to determine whether the experimental subjects responded to either volatile or nonvolatile chemicals, as they could detect both volatile and nonvolatile chemicals close to bedding by poking their noses into the soiled bedding. Even when using filter paper soaked with stimulus urine, preventing direct contact with the filter paper is important for determining stimulus attributes, such as volatile vs. nonvolatile [48,49]. In addition, if a highly volatile chemical is a critical attractant, the time between soiled bedding or urine sample collection and presentation and preservation is important.

The attractant for opposite-sex conspecifics may not be released only in the urine. In fact, the olfactory preference apparatus introduced above presented the odor emitted from awake stimulus animals through airflow to subjects [6]. During the test, stimulus animals sufficiently acclimated to the apparatus did not always excrete urine and feces, but rather emitted their body odors. Some studies used anesthetized animals as stimuli for the preference test [45,50]. In fact, MUPs transporting volatile pheromones are not only produced in the liver and excreted from the kidney, but also expressed in exocrine glands such as the mammary, lacrimal, parotid, sublingual, and submandibular glands [51,52]. It is possible that secretions from these glands warmed by body temperature result in pheromone volatilization, and consequently become an attractive odor for opposite-sex conspecifics.

Our study found that when the odors of castrated and gonadally intact males were simultaneously presented in the preference test, sexually active male rats preferred that of the castrated one [53,54,55]. We considered it unlikely that the lack of testosterone (T) due to castration may newly produce an attractant for males, but that the lack of T disinhibits the negative feedback of the hypothalamus-pituitary-gonadal (HPG) axis, resulting in increased circulating levels of gonadotropin-releasing hormone (GnRH) and gonadotropins, and consequently, that those became male incentive odors. Therefore, we examined the effect of GnRH antagonist administration to castrated male rats, and found that the castrated male attractiveness decreased to levels comparable to gonadally intact males [56]. Furthermore, we compared the responses of sexually active male rats to the odors of castrated males with and without surgical removal of the pituitary gland (hypophysectomy, HPx) to remove the source of gonadotropin, demonstrating longer exploration of the odor of pituitary-intact mice than that of HPx males [56]. However, the odor of HPx males was still more attractive than that of gonadally intact males, indicating that the attractiveness of castrated males is not due only to elevated gonadotropin levels but also to increased GnRH. Furthermore, we examined the effect of administration of human and equine chorionic gonadotropins (hCG and eCG) on luteinizing hormone (LH) and follicle stimulating hormone (FSH) action in the rat, respectively; in HPx males, the recovery of attractiveness followed eCG, but not hCH, injection [56]. We thus concluded that the attractiveness of castrated males to sexually active male rats is due to elevated levels of both GnRH and FSH due to the lack of circulating T.

Male rats attracted to the odor of castrated males must be primed with estrogen or aromatizable androgens because males treated with aromatase inhibitors or castrated males treated with nonaromatizable dihydrotestosterone (DHT) did not prefer castrated males [57], indicating that the odor of castrated males must be explicitly different from the estrous odors attracting castrated males treated with not only estrogen but DHT. In real ecological environments, however, there is no opportunity for castrated male odor to attract sexually active males. Nevertheless, the existence of neurons specifically activated by castrated male odors in the mouse AOB has been reported [58,59]. Furthermore, glomerular activity maps for sexually mature female mouse urine overlapped with maps for gonadectomized urine of both males and females [59]. Our studies, therefore, suggest that the production of this attractive odor also occurs in females during estrus evoked by the surge of GnRH and gonadotropins. In the future, identifying the molecules responsible for the attractiveness of castrated males (also of estrous females) needs to be investigated.

## 4. Sexually Dimorphic Preference and Organizational Effects of Sex Steroids

As shown in Figure 1B, preferences for conspecific odors are distinctly sexually dimorphic; sexually mature active males prefer the odor of receptive females to that of males or ovariectomized anestrous females, while receptive females prefer the odors of sexually active males to that of females or castrated sexually inactive males [6]. These sex-specific preferences are due to sex differences in the brain resulting from the organizational effects of perinatal sex steroids, as administration of opposite-sex hormones after gonadectomy in adult animals could not reproduce a heterosexual preference pattern [57,60,61].

Rodents with a short gestation period, such as rats and mice, have the critical (sex-steroid sensitive) period for brain sexual differentiation during the perinatal period. Neonatal castration in male rats significantly reduced the preference for receptive females, recovered by consequent treatment with testosterone propionate (TP) but not DHT propionate [62]. In addition, administration of the antiestrogen nitromifene citrate to pregnant rat dams eliminated the adult masculine preference in male offspring, whereas prenatal administration of an antiandrogen, cyproterone acetate, had no effects [63]. Furthermore, male rats neonatally injected with the aromatase inhibitor 1,4,6-androstatriene-3,17-dione (ATD) also showed decreased preference [4,5,64,65], which was more effective than prenatal ATD administration [4]. These results, together, indicate that estrogenic action during the critical period in early development is important for masculinization of sexual preferences in the rat. However, exposure of gonadally intact male rats to excessive androgen appears counterproductive, as TP embryonic administration (ED17–19) to pregnant dams has been reported to produce adult male offspring who spend longer times with stimulus males rather than estrous females [66]. Additionally, chronic TP administration to male pups by subcutaneous implantation of a Silastic capsule containing TP after birth did not affect the time spent with females, but increased the time spent with males in adult preference tests, resulting in a loss of sexual preference [67].

On the other hand, sex hormones are not necessary for the normal development of the female brain. Postnatal chronic estradiol benzoate (EB) administration to female rat pups during lactation results in male preferences, i.e., preferring estrus females to males after sexual maturity [68]. Contrarily, pubertal sex hormones seem to promote female preferences. It has been reported that although T treatment in adult ovariectomized females did not lead to any sexual preference, chronic estrogen administration during postnatal days 30 to 90 made ovariectomized females show female preference after T treatments [69] (in our study, though, ovariectomized females showed female preference by implantation of a Silastic capsule containing T [6]).

In mice deficient for the estrogen receptor-α gene (ERα [70]), males decreased investigation time for estrous females in the preference test using anesthetized males and females as stimuli, which resulted in abolishing sexual preference. In contrast, in mice deficient for the estrogen receptor-β (ERβ [71]) gene, the males showed a normal level of masculine preference comparable to that of WT mice. Further, aromatase-KO (ARKO) male mice lost their preference for both volatile and nonvolatile odors of estrous females [72]. According to these results, it can be considered that the estradiol (E2) converted from circulating T secreted from the male testes by the aromatase expressed in the mouse brain acts on ERα –similar to rats– to masculinize the male brain and promote the display of male-typical preference.

On the other hand, there is evidence of the involvement of androgen receptors (AR) in the sexual differentiation of sexual preference. Tfm (the androgen-insensitivity strain, named from the human syndrome, “testicular feminization”) males made longer nose contact to male cage bedding than to estrous female bedding, and showed no preference in a Y-maze test where the goal boxes had sexually active male or receptive female stimulus mice [73]. Another study demonstrated that female mice injected with DHT at birth showed normal feminine sexual behavior but masculine preference in adulthood, i.e., spending longer time in the investigation of estrous soiled bedding and staying in the vicinity of estrous females rather than males, while E2 injection at birth failed to masculinize the adult preference for soiled bedding [46]. However, conditional ARKO male mice in the nervous system sparing hypodevelopment of the reproductive organs, preferred to investigate female rather than male soiled bedding, like WT males [74]. Together, the differentiation of mouse sexual preferences may be affected by both ER and AR, and the difference might be minute, such that masculinization might be affected by ER and feminization by AR.

In rodents, it has been considered that sex hormones, especially estrogen, are not required for female brain differentiation to display feminine preference. However, compared to WT females, ARKO female mice showed shorter sniffing time investigating both male and female volatile odors, resulting in no preference [50]. Later, it was found that progesterone (P) receptor expression transiently appears in the anteroventral periventricular nucleus and medial part of the medial preoptic nucleus, both important brain regions for sexual preference, during postnatal days 15 to 25 in normal female mice, whereas those regions in ARKO females did not express the P receptor during that period [75]. Therefore, they examined the effect of prepubertal EB treatment in ARKO females, demonstrating that estrogen action during postnatal days 15 to 25 plays an important role for the normal development of sexual preference in female mice [76]. In other words, estrogen might achieve brain masculinization and defeminization during the fetal period, and conversely promote feminization of the brain after birth but before puberty.

## 5. Hormonal Control for Opposite-Sex Preference

Because preference for opposite-sex chemosensory signals is measured by an approaching behavior toward sexual partners, a part of mating behavior, it depends—as with sexual behavior—on circulating sex steroids. Female sexual behavior strictly requires estrogen binding to ERα in the brain and is enhanced by the P action following estrogen [77]. On the other hand, it is well established that estrogen, converted from circulating T in the brain, is also critical for male sexual behavior, especially in rodents. Male mice deficient for ERα [78,79] or aromatase [80] show severely impaired intromission behavior, although the penile functions, both reflexive and noncontact penile erection [79,81], require AR for activation [82,83,84,85].

**Figure 2:**
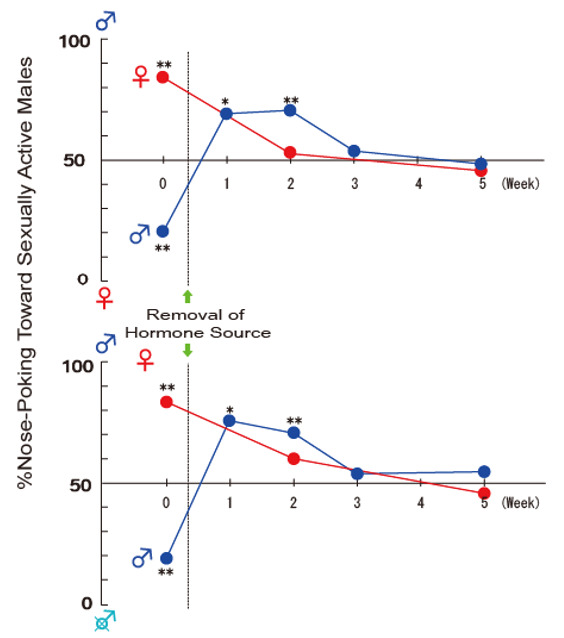
Effects of sex hormone ablation on sexual preference. The results in preference tests between sexually active male vs. estrous female and between sexually active male vs. castrated male are shown in the upper and lower panels, respectively. Ovariectomized female rats were primed with estrogen and progesterone in the preference test at week 0, and thereafter tested without sex hormone treatment. Male rats were gonadally intact and castrated immediately after the preference test at week 0. Male rats transiently displayed the feminine type of sexual preference during 2 weeks after castration [6].

Similar to endocrine controls of sexual behavior, adult male rat castration eliminates masculine preference, which can be restored by T replacement therapy [6,62,64]. On the other hand, chronic treatment, but not a single injection [64,86], of either estrogen or DHT can induce masculine preference in castrated male rats [57]. Interestingly, male rat castration induces transient feminine preference, preferring male odors over estrous odor for one to two weeks followed by disappearing preference [6,57], while removal of the sex hormone source in female rats does eliminate sexual preference (Figure 2).

**Figure 3:**
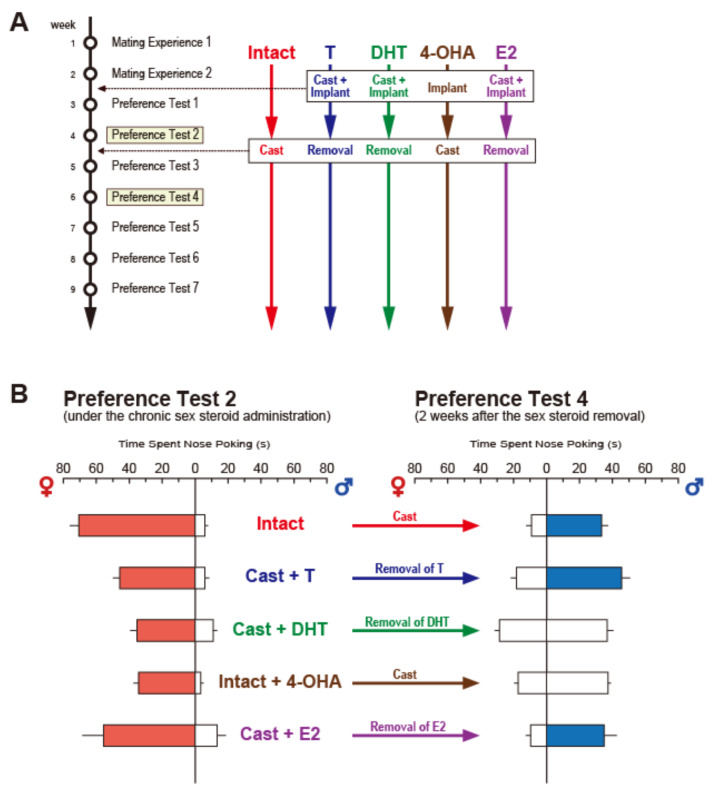
We examined the hormonal influence on the transient reversal of sexual preference observed after castration in male rats [57]. (**A**): in the study, all male rats received mating experience sessions twice weekly followed by seven preference tests. The intact group was castrated after the second preference test; T, DHT, and E2 groups were castrated a week before the first preference test and simultaneously implanted with Silastic capsules containing T, DHT, and E2, respectively. 4-OHA group received a subcutaneous implantation of a Silastic capsule containing a steroidal aromatase inhibitor, 4-OHA, a week before the first test and were castrated after the second preference test. (**B**) shows only the results in preference tests 2 and 4 as the most important points in this experiment. The DHT and 4-OHA groups showed a lack of estrogen after treatment, resulting in no reversed preference after steroid supply ablation. Colored bar in each column of (**B**) indicates significantly longer time in nose-poking toward females than males (red) or toward males than females (blue) (*p* < 0.05). Reprinted with permission from Ref. [57]. Copyright 2015 Elsevier.

In order to examine which receptors, ER or AR, mediate this transient reversal of preference following castration, we prepared five experimental groups, each by two steps of hormonal manipulations: Intact group, first gonadally intact and then castration; T group, first castration and T implantation (subcutaneous implantation of a Silastic capsule containing T) and second, capsule removal; DHT group, first castration and DHT implantation and second, capsule removal; 4-OHA group: first gonadally intact and 4-OHA (4-hydroxyandrostenedione, steroidal aromatase inhibitor) implantation and second, castration; and E2 group, first castration and E2 implantation and second, capsule removal (Figure 3A). The olfactory preference tests were carried out twice after the first step and four times after the second [57]. In the preference test after the first step, all males displayed male-typical olfactory preferences, preferring estrous odor to male or anestrous odor, and castrated male odor to sexually active male odor. Following removal of the hormonal sources as the second step, males in Intact, T, and E2, but not DHT and 4-OHA, showed reversed female-typical preference, preferring male odor to estrous or castrated male odor, within 2 to 3 weeks after the second step (Figure 3B). Based on these results, we concluded that the transient reversal of sexual preference is mediated by ER, activated by estrogen, and converted from declined circulating T following castration [57].

**Figure 4:**
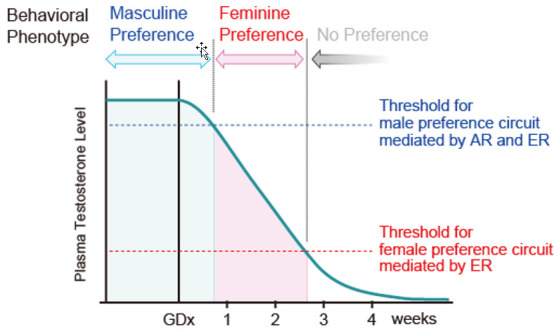
Model explaining the transient reversed preference observed after male rat castration. We hypothesize that the male rat brain has both circuits for expression of masculine and feminine preference. In gonadally intact males, their circulating testosterone is sufficient to activate the masculine circuit, displaying dominant masculine preferences, and obscuring feminine preferences. However, the declining level of circulating testosterone after castration becomes insufficient to activate the masculine circuit, while still activating the feminine circuit because of its lower threshold in steroid sensitivity, therefore leading to a temporal feminine preference as the level falls below the threshold.

The model to explain this phenomenon is shown in Figure 4. This model assumes neural substrates for both masculine and feminine preferences in the rodent brain, and that masculine substrates have a much higher activation threshold by sex hormones via AR and ER than the feminine. In normal physiological states in males, the circulating androgen level is sufficiently high for activating the masculine substrate, prevailing over the feminine, and consequently determining the masculine preference phenotype. However, declining androgen levels after castration become insufficient to activate the masculine substrate but are still sufficient for the feminine substrates, making feminine preference the temporal phenotype, with both disappearing thereafter following further decreases in the androgen level. Chronical implantation of E2 capsules [57] or daily E2 injections [60] in castrated males increase the circulating androgen level beyond the masculine substrate threshold, consequently leading to a masculine preference, whereas a single E2 injection, insufficient for masculine but sufficient for feminine substrates, induces feminine preference in castrated males [6]. This model is also supported by the finding that semaphorin 7A gene deletion, which prevents migration of GnRH neurons born in the olfactory placode into the brain, resulting in hypoandrogenism, led to male mice showing feminine preference [87].

In female rats, the sexual preference for male volatile odors changes with estrous cycle, i.e., the preference is higher in the proestrus/estrus phase than in the diestrus phase, and diminishes with pregnancy [88]. This suggests that estrogen plays a critical role in the female preference for male odors, though chronic EB administration [1] or an E2 and P combination treatment [6,89] is required for a clear preference. In contrast, female rats showed preference for male soiled bedding independent of sexual experience and circulating sex hormones [90]. On the other hand, it has been reported that administration of 0.4 mg TP three times a week was insufficient for clear preference in female rats [1], whereas chronic T treatment by a Silastic capsule >2 weeks induces feminine preference [6,61]. In addition, chronic administration of a nonaromatizable androgen, DHT, and a synthetic and orally active anabolic androgen, methyltrienolone, also induce preference in female rats, indicating that the preference for male odors in female rats can be induced not only by ER activation but also potentially by AR activation [61].

As mentioned previously, female rat preference greatly depends on whether females are allowed to have direct mating with stimulus males [2]. This may be due to aversiveness of vaginal stimulation by males [1,2], but may be also affected by a female trait, the preference to stay with other females [91]. In mice, although estrous females prefer the volatile odors of sexually active males to those of females [53], virgin females show preference for female rather than male soiled bedding [92], having no aversive factor. Furthermore, the preference of female hamsters for male odors does not depend on sex hormones [93]. Further detailed analyses are needed for the elucidation of hormonal regulation in the feminine preference for male odors with consideration for species specificity, test conditions, mating experience, etc.

## 6. Regulation of Opposite-Sex Preference by Integrating the Main and Accessory Olfactory Systems

As mentioned previously, the rodent nasal cavity has mainly two chemosensory organs, the OE and the VNO. Because the latter is known as a pheromone receptor, extensive research has been carried out on the VNO function in a variety of social behaviors [94,95]. Although surgical VNO removal (VNOx) exerts a severe impact on sexual behavior in sexually naïve male hamsters [96], the effects on sexual behavior in rats [97,98,99] and mice [100,101] are weak and limited.

In contrast to sexual behavior, VNOx in female mice abolished the preference for male soiled bedding and decreased lordosis quotient (%displays of lordosis to male mounts), though it had no influence on the preference for volatile body or urine odors of males [45]. It also confirmed the ineffectiveness in the preference for airborne estrous female body odor in male rats [99]. On the other hand, TRPc2 gene deletion, which causes dysfunction in vomeronasal receptors, led to male mice displaying sexual instead of aggressive behavior toward males [102] and eliminated the preference for estrous female bedding [103,104]. Furthermore, female TRPc2-KO mice showed vigorous mounts and male sexual behavior, regardless of males and females [105]. However, surgical VNOx in female mice failed to promote mount behavior toward males [106]. This discrepancy in the effect between the gene KO and the surgical operation may be due to the broad distribution of the TRPc2 gene, densely expressed in vomeronasal neurons, but sparsely in erythroblasts, sperm, and the brain [107]. Dysfunction not only in the VNO but other central nervous activity caused by TRPc2-KO may have a different influence on sexual behavior.

OE destruction by zinc sulfate (ZnSO_4_) infusion into the nasal cavity did not completely eliminate male rat sexual behavior [99], but severely impaired male mouse sexual behavior [108] and female mouse lordosis [109], as well as eliminated the preference for volatile body odors [99,108,109] and for odors of directly contactable urine [108,109]. Furthermore, male mice with olfactory neuron loss, specifically in the dorsal OE and produced by targeted depletion of the cyclic nucleotide-gating olfactory channel combined with the diphtheria toxin fragment-A, had normal sniffing behavior and pheromonal detection but no preference for female urine [110]. In addition, ablation of α subunit of G_O_ expressed in OE olfactory receptors induced structural changes in the MOB by reduced neurogenesis and increased cell death, subsequently decreasing the number of calbindin-positive interneurons and increasing that of thyrosine hydroxylase-positive interneurons in the MOB. As a result, male mice showed no preference for female soiled bedding and reduced anogenital investigation during mating, but still maintained the ability of food-finding [111]. In the rat, some males show no sexual behavior even after several mating sessions in a certain probability, interestingly; they have an olfactory deficit other than social odors and show no sexual preference [112,113].

Signals in the OE activate mitral cells in the MOB, whereas signals detected in the VNO activate mitral cells in the AOB. Optogenetical inhibition of mitral cells in the AOB significantly suppressed lordosis in ovariectomized, but EB and P primed, female mice, indicating that pheromones in the VON facilitate female sexual behavior in female mice [114]. In addition, male mice with lesions in the AOB showed a significant decrease in the time spent investigating estrous females, but still could discriminate between estrous and anestrous female odors or between male and female odors [115].

While the main and accessory olfactory systems have a parallel relationship in the projections, their interaction does not appear simple. ZnSO_4_ lesions in female OE mice prevented the increase of cFos-positive neurons in the MOB by exposure to the volatile odors in male urine but not in the AOB [109,116]. Conversely, VNOx suppressed increased cFos expression in AOB mitral cells after sexual behavior but not in the MOB [98]. Recently, it was shown that neurons in the MOB and the AOB have a complicated crosstalk in the bulb [117]. On the edge of the rostral AOB, i.e., between the dorsal part of the MOB and the AOB, there is a structure called the olfactory limbus, which receives afferents from a variety of OE receptors including TAARs. The olfactory limbus function is not yet properly characterized, but these input signals are likely aggregated to the large principal cells in the AOB and sent to the medial nucleus of the amygdala (MeA), which plays a critical role in social chemosensory signal processing [118].

It is also possible that the mutual interaction between the MOB and the AOB is mediated by the MeA, the secondary olfactory region. Optogenetic stimulation of mitral cells in the male mouse AOB expressing channelrhodopsin, and induced by Cre recombinase co-expressed with protocadherin 21, led to activation of main stream projection from the AOB to the MeA, and increased nose-poking to estrous female urine but not to male urine [119]. On the other hand, neurons in the MeA also receive afferent inputs from the MOB. Stimulation by volatile male urine odor in female mice induced cFos expression in mitral cells in the MOB, which send axons directly to the MeA [120]. Furthermore, injection of a retrograde neural tracer, cholera toxin B, into the AOB revealed that AOB neurons receive direct innervation from the MeA [121]. Mitral cells in the posteroventral MOB, which expresses an axon guidance molecule, Neuropilin 2 (Nrp2), send attractive social signals to the MeA. Mitral cell-specific Nrp2-KO abolished preference for estrous female urine in male mice and induced a loss of preference for castrated male urine containing female-attracting pheromone, (methylthio)methanethiol [122,123], in female mice [124].

**Figure 5:**
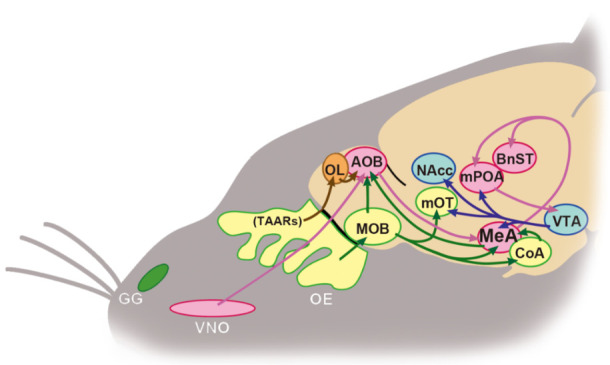
Schematic diagram of supposed chemosignals that modulate preference for opposite sex in mice. Red, green, brown, and blue arrows represent vomeronasal, main olfactory epithelial, TAARs, and DA rewarding signal streams, respectively. OE: olfactory epithelium, VNO: vomeronasal organ, GG: Grueneberg ganglion, TAAR: trace amine-associated receptor, MOB: main olfactory bulb, AOB: accessory olfactory bulb, OL: olfactory limbus, mOT: medial olfactory tubercle, CoA: cortical nucleus of amygdala, MeA, medial nucleus of amygdala, BnST: bed nucleus of stria terminalis, mPOA: medial preoptic area, VTA: ventral tegmental area, NAcc: nucleus accumbens.

Thus, it has become clear that the MeA, aggregating the main and accessory olfactory systems, plays an important role in the regulation of opposite-sex odor preference (Figure 5). A report comparing the effect of lesions in the anterior and posterior parts of the MeA in female mice demonstrated that both lesions severely suppressed lordosis, while only the posterior lesions eliminated preference for volatile and nonvolatile male urine odors [48]. In addition, silencing of MeA neurons by designer receptor exclusively activated by designer drug (DREADD) in female mice abolished the preference for volatile and nonvolatile male odors [125]. On the other hand, aromatase-positive MeA neurons receive afferent projections from the anterior part of the AOB, which relays pheromonal signals from V1R in the VNO. This projection from the AOB to the MeA is sexually dimorphic, much thicker in males than in females [126], and MeA neurons distribute social, sexual, and predator signals from the AOB to other regions, including the hypothalamic nuclei, in a sex-dependent fashion [127]. Dopamine receptor D1R-positive neurons in the posteroventral part of the MeA respond to both social and predator signals that mice should approach or avoid, respectively. When receiving dopamine (DA) signals from the midbrain ventral tegmental area (VTA), these D1R-positive neurons activate neurons in the bed nucleus of the stria terminalis (BnST), consequently leading mice to approach to the odor, whereas without DA signals from the VTA, these neurons activate others in the ventromedial hypothalamus, resulting in odor avoidance [128].

In contrast to this vomeronasal signal processing in the MeA, odor signals that drive innate behavior traveling via the MOB are conveyed to the cortical nucleus of the amygdala (CoA), and similarly to the MeA, sorted by whether to approach or avoid [129]. Optogenetic suppression of the projection form the MOB to the CoA lead to male mice that no longer avoid predator odors, and eliminated the approach to 2-phenylethanol, an innate mouse attractant [130]. In addition, simultaneous electrophysiological recordings of neuronal activities in the olfactory bulb and the amygdala in freely behaving female mice demonstrated synchronicity between the olfactory bulb and the MeA during male urine investigation, whereas synchronicity was found between the olfactory bulb and the posteromedial cortical amygdala (CoApm) during female urine investigation [131].

In male hamsters, a CoApm lesion has been reported to alter female investigation during mating but not to affect the preference for volatile odor of female bedding [132]. After injection of retrograde tracer, cholera toxin subunit B (CTB), into the anterior part of the MeA (MeAa) or the posterodorsal part of the MeA (MeApd) in male hamsters, activation of these labeled cells by cFos was examined when the injected animals were placed in a vacant female or male cage, suggesting the integration of social signaling in the MeAa and steroidal signaling in the MeApd [133,134]. Furthermore, combined unilateral lesions in the MeAa and MeApd impaired the preference for volatile odors in male hamsters when lesions were located contralaterally, but not ipsilaterally [134]. It should be noted that destruction of the nucleus of the lateral olfactory tract, a part of the MeAa, in male rats abolished avoidance of predator odors, decreased sexual behavior, and eliminated preference for estrous females [135].

In the neural regulation of male rat sexual behavior, estrous signals are transmitted to the MeA, processed, and transmitted to the BnST and medial preoptic area (mPOA) through the stria teminalis [136,137]. On the other hand, in male hamsters, combined lesions in the unilateral MeA and the contralateral mPOA impaired sexual behavior but not affected sexual preference, whereas combined lesions in the unilateral MeA and the contralateral BnST failed to suppress sexual behavior but did impair preference for volatile, but not nonvolatile, female odors [138]. These results suggest the importance of the BnST, more than the mPOA, in male hamster olfactory preference regulation. Indeed, bilateral lesions in the posterior BnST eliminated the preference for opposite-sex odors [139]. In contrast, mPOA lesions [140,141] or inactivation [142] completely suppressed olfactory preference for females in male rats, suggesting species-specific regulation of sexual preference even within rodents.

In female rats [143] and female hamsters [144], in contrast to males, mPOA destruction eliminated sexual preference for males. In female mice, mPOA neurons projecting to the VTA express neurotensin [145]. In response to male urine odor and E2, these neurons promote DA release in the nucleus accumbens (NAcc) [144], which plays an important role in motivational behavior as a part of the brain reward system. Indeed, DA release in the NAcc in sexually naïve female mice led to preference for male soiled bedding [146]. The medial olfactory tubercle (mOT), possibly involving reinforcement of odor learning and hedonic processing in drug abuse, is as another reward system driven by opposite-sex odors. The mOT receives direct inputs from not only the MOB [147] but also the MeA [148,149], and mOT lesions abolish preference for male bedding in female mice [150]. Following CTB injection into the female mouse mOT, exposure to male bedding showed a significant increase of cFos expression in MeA neurons and VTA neurons projecting to the mOT, whereas DREADD silencing in the mOT interrupted the preference for opposite-sex chemosignals in female mice [49].

On the other hand, serotonergic (5-HT) neurons in the midbrain raphe nuclei may also be involved in sexual preference. Male mice deficient in 5-HT neurons by co-expressing Cre with dPet1, a marker gene for 5-HT, combined with the floxed Lmx1b allele showed no preference for estrous female odor [44]. This was also confirmed in mice deficient for tryptophan hydroxylase 2, a synthetase mediating the first step of 5-HT biosynthesis [44]. Furthermore, female mice with 5-HT neuron loss by floxed Lmx1b showed transformed sexual preference, i.e., from estrous odors to male odors, and increased mounts toward receptive females [151]. However, there is a contradictory report that Tph2-KO male mice exhibit a normal masculine preference [152]. Because 5-HT neurons in the midbrain raphe nuclei have projections to the broad area in the whole forebrain with abundant types of receptors, exactly 14 including subtypes, each with its own characteristic function, a complete abolition in all 5-HT systems may have situation-dependent multiplex effects.

## 7. Experience and Sexual Preference

Male reproductive activity is strongly dependent on sexual experience, including sexual behavior. More than half of sexually naïve male rats, although interested in receptive females in the first mating session, would not accomplish a complete copulatory behavior. However, following weekly mating sessions, most males would begin sexual activity soon after the introduction of females. Such experience dependency is also observed in the noncontact erection of male rats, induced by estrous female odor [153]. Early studies showed that male rats without sexual experience show no preference for urine odors of estrous females [154], even though the pheromonal effect is an innate response [155]. In contrast, male mice [43,156] and male hamsters [157,158] show a preference for estrus female odor without sexual experience, although the gradual increase in time spent in olfactory investigation depends on sexual experience [53]. In hamsters, sexual experience affects the performance of sexual behavior after minor damage to the mPOA [159]. Herein, we attempt to discuss the effect of experience on preferences for opposite-sex odors in this section.

The olfactory environment during housing may exerts some influence on OE neurons. Sex-separated housed males from weaning to 6 months old exhibited more expressed genes in the OE, and a subset of these chemoreceptors showed altered expression frequencies depending on sex-separation and olfactory deprivation [160]. In addition, olfactory memory is formed at the olfactory bulb level, with estrogen facilitating odor learning in the MOB [161]; simultaneous olfactory inputs and noradrenaline release from the terminals of locus coeruleus neurons bring specific and long-lasting changes in the MOB, exerting influence on the preference for foods and urine odors [162]. In male mice, mating experience increases the densities of newborn cells in the AOB granule cells [163]. Furthermore, cFos expression in the piriform cortex, receiving direct projections from the MOB, coincided with sexual preferences before and after mating experience [164]. A study observing activities in mouse MeA neurons for several months by in vivo calcium imaging demonstrated that the MeApd exhibits persistent sex-specific changes mediated by oxytocin [165].

Sexual experience may exert physiological changes also in the mPOA. As mentioned above, excitotoxic lesions in the mPOA abolished the volatile odor preference of sexually inexperienced male hamsters, but had no effect on experienced males or nonvolatile chemosignals [159]. In mPOA, furthermore, nitrogen oxide synthase (NOS) expression in male rats [166] and AR, but not ERα, in male mice [167] increased by sexual behavior. Recently, Vgf gene expression was found to be increased in the rat mPOA including the sexually dimorphic nucleus of the preoptic area (SDN-POA) after sexual experience, while local Vgf knockdown in the mPOA by adeno-associated viral vector prevented experience-dependent enhancement of sexual preference [168]. Although the function of the Vgf gene is not well known at present, Vgf protein and its derived peptides are secretory and involved in reproductive functions, such as energy metabolism and penile erection [169,170], so further research is expected to clarify the relationship with sexual function and experience.

In addition to the innate and physiological effects of sexual experience described above, preference might be acquired and enhanced via conditioning. Sexually naïve male mice [43,156] and hamsters [171] can learn conditioned place preference (CPP) through exposure to estrous urine and bedding, meaning that those stimuli have a rewarding value. In male mice, CPP for such sexual stimuli could be acquired even after blocking vomeronasal inputs by surgical VNO removal [156], by lesion in the AOB, or after blocking olfactory epithelial inputs by ZnSO_4_ lesion in the nasal cavity, but not after blocking both [43]. When a male mouse encountered another male and estrous female, sexually naïve WT males attempted to investigate and mount the females, whereas TRPc2-KO mice showed comparable investigation and mounts toward both males and females [104]. At this time, DA release was found to increase in the NAcc of WT, but not TRPc2-KO mice [104]. Such activation of the reward system by DA may produce association learning with simultaneous sensory inputs, further reinforcing sexual preference [172,173].

In rat studies, sexually naïve males displaying no sexual preference showed not only increased cFos expression [174,175] but also increased DA release [176] in the NAcc in response to untouchable estrous females or estrous female bedding. Gaining sexual experience, however, significantly increased the number of cFos-immunoreactive cells [175] and DA release [176] in the male rat NAcc.

On the other hand, female mice, if able to directly contact male bedding or urine, exhibited preference for such nonvolatile male chemosignals without sexual experience, while if not able, they showed no preference for such male odor [177,178,179]. However, with repeated experience of direct contact with male bedding or urine, sexually inexperienced female mice started to show preference for volatile male odors [177,178,179]. In addition, sexually naïve females failed to acquire conditioned incentives by exposure to nonvolatile male odors in diestrus or after ovariectomy, implying its sex hormone dependency, whereas sexually experienced females successively acquired such conditioning even after ovariectomy, implying sex hormone independency [180]. Exposure to primary pheromones attracting sexually naïve female mice activates the basolateral nucleus of the amygdala (BLA) and the NAcc shell but not the VTA and the orbitofrontal cortex, whereas that of secondary attractants acquired via sexual experience activates not only the BLA but also the prefrontal cortex and the VTA [177]. Notably, the BLA role in connecting innate and learned attractants deserves attention.

In contrast to female mice, sexually inexperienced female rats show preference for male bedding consisting of both volatile and nonvolatile chemosignals [146,181,182] and for airborne male body odors [181]. Although exposure to male bedding in sexually inexperienced female rats did not change the number of cFos-immunoreactive cells in the NAcc [181], the increased DA release in the NAcc following male bedding stimulation was detected by microdialysis, and prevented by administration of a glutamate antagonist, resulting in decreased time spent investigating male bedding [146]. At present, where the neurons releasing DA in the NAcc project from is not known, but one such region may be the mOT. As mentioned in the previous section, because the mouse mOT receives direct innervation from the MOB and activates the reward system, it should be studied whether the rat mOT also involves in the mediation between chemosensory inputs and the reward system as well.

It is not that sexual experience has no effect on sexual preference in female rats. Following mating sessions with sexually active males where scented objects (a rubber duck with orange extract or a rubber bone with almond extract) were placed, female rats showed conditioned preference for those objects [183]. Namely, sexual experience acquires a reward value in sexual behavior, which makes possible associative odor learning. In addition, administration of selective androgen receptor modulator [9-chloro-2-ethyl-1-methyl-3-(2,2,2-trifluoroethyl)-3H-pyrrolo-[3,2-f]quinolin-7(6H)]-one after ovariectomy effectively induces sexual preference in sexually experienced, but not sexually naïve, female rats [184]. Furthermore, although no difference between sexually experienced and naïve female rats in cFos expression in the mPOA by sexual behavior was found, the increase in NOS expression was only detected in the mPOA of sexually experienced females [182]. Thus, sexual experience may induce physiological changes in female rat brain, affecting sexual preference.

## 8. Neuropeptidergic Regulation of Opposite-Sex Preference

Vassopressin (AVP) and oxytocin (OT) are posterior pituitary hormones, as well as neuroregulatory peptides, expressing in the main and accessory olfactory systems, the limbic systems, hypothalamic nuclei, etc. [185,186,187,188], and regulate a variety of olfactory-dependent social behaviors. For example, mice deficient for the OT gene showed a lack of social recognition [189,190,191], while mice deficient for the vasopressin receptor 1b (V1bR) could poorly discriminate familiar and novel females compared to WT mice [192].

Regarding the effect of preference for opposite-sex odor, the results seem to differ depending on stimulus presentation. In a three-chamber apparatus with side chambers having wire-mesh caged stimulus males or estrous females, WT male mice spent time in the female chamber longer than in the male chamber, whereas males deficient for the OT gene showed neither preference regardless of direct contact with the stimuli [53]. In contrast, OT-KO female mice did show a normal preference for male bedding as WT females [53]. However, another study reported that both male and female OT-KO mice exhibit a loss of preference for the odor of male and estrous female bodies [53]. In addition, conditioned oxytocin receptor (OTR)-KO in aromatase-positive neurons in the MeA abolished sexual preference in male mice [53], suggesting that chemosensory signals in the VNO and OE regulating sexual preference are processed in aromatase-positive oxytocin-sensitive neurons in the MeA. Pharmacological studies also demonstrated OT involvement in sexual preference. Oral administration of a non-peptide oxytocin antagonist in male mice impaired sexual preference, but did not affect social preference or social novelty preference [193]. In male rats, intraperitoneal injection of the above non-peptide oxytocin antagonist did not affect sexual preference per se, but significantly reduced time spent in investigation for estrous females [194].

OT also seems to enhance responses to social chemosensory signals via reward system activation. In estrogen-primed ovariectomized female mice, intracranial OT injection, although of little rewarding value, may have enhanced rewarding value if combined with social stimuli [195]. Male rats with intracranial injection of either OT or quinpirole, a D2 agonist, during repeated co-habituation with another male scented with almond extract came to prefer the scented male rather than estrous females [196]. These suggest a critical role of OT in the acquisition of attractive odors via the rewarding system.

On the other hand, there are not many studies on AVP in the regulation of sexual preference. Male mice with double depletion of two AVP receptor genes, V1aR and V1bR, showed a normal preference for estrous female bedding rather than that of males [197], and more preference for estrous body odor than that of males, comparable with that of WT males [197]. Nevertheless, the possible involvement of AVP in sexual preference may not be excluded, because of a report that male rats perinatally treated with ATD, an aromatase blocker, significantly increased the number of AVP-positive cells in the suprachiasmatic nucleus and became bisexual in the partner preference test [198]. In addition, WT and V1bR-KO male mice were subjected to preference tests with clean bedding vs. soiled bedding, revealing that the investigation time in WT males was higher for estrous female bedding than for that of males and higher for male or estrous female bedding than for a clean one, whereas V1bR-KO mice showed no preference at all [199]. Furthermore, it has also been reported that vasopressinergic projections from the MeA and BnST to the ventral pallidum (VP) in the rat are sexually dimorphic, much denser in males, and that local application of a V1aR antagonist into the VP impaired male preference for estrous females, but conversely enhanced female preference for males [200]. Further investigation is necessary to elucidate the involvement of the forebrain AVP system in the regulation of preference for opposite-sex odors.

There is another line of studies reporting that kisspeptin, which mediates between sex hormones and GnRH neurons in the HPG axis, is involved in the regulation of sexual preferences. Female mice exposure to male bedding or urine activated kisspeptin-immunoreactive cells in the rostral periventricular area of the third ventricle (RP3V) [201], and this activation was completely suppressed by VNO surgical removal but not by ZnSO_4_ lesion of the OE [202]. In fact, kisspeptin gene deletion abolished preference for male odors, while a compensatory treatment with kisspeptin recovered the preference [202]. Because RP3V kisspeptin neurons have direct projections to GnRH neurons, genetic disruption of Dicer in GnRH neurons, abolishing GnRH expression in GnRH::Cre; DicerloxP/loxP female mice produced a female-directed preference, but did not affect lordosis [202]. RP3V kisspeptin neurons have another downstream path, projecting to neuronal NOS (nNOS) in the ventrolateral part of the ventromedial hypothalamus (VMHvl). nNOS-KO female mice did not show a preference for male odors, while peripheral injection with a cocktail of NO donor and soluble guanylate cyclase agonist in nNOS-KO females recovered this preference for male odor [202].

On the other hand, kisspeptin may function in the regulation of male preference; specific activation of kisspeptin neurons in the MeApd by DREADD significantly facilitated investigatory behavior for estrous females during preference tests [203]. Local infusion of kisspeptin into the MeApd increased circulating LH and spontaneous penile erection, while infusion in the dorsal part of the MeApd produced only an increased level of circulating LH [204], suggesting that kisspeptin in the MeApd regulates sexual behavior separately to mediating the HPG axis. Presumably, the neural regulation of sexual preference by kisspeptin may be independent of its role in the HPG axis.

## 9. Future Outlook

In this review, we covered the available studies on the physiological mechanism of preference for chemosensory signals, which plays an important role in the social behavior of small rodents. This preference may be affected by several internal factors. That is, if animals show no preference at all when simultaneously presented with male and estrous female stimuli, the animals may have declined sexual motivation, lose the incentive value for the stimuli, or not be able to discriminate or detect those stimuli. The findings reviewed here include some requiring further analysis to determine which of these factors contribute to the result. In addition, we limited our review to the preference for chemosensory signals, but small rodents utilize other sensory modalities, such as ultrasonic vocalization, for courtship in mating. Social behavior in animals, including humans, is extremely complicated; therefore, further research is needed for their comprehensive understanding.

The sexual preference addressed here contains many elements as characteristics specific to each sex. This means that sex should be understood not as a dichotomy, but as a spectrum.
